# Affordability of essential medicines: The case of fluoride toothpaste in 78 countries

**DOI:** 10.1371/journal.pone.0275111

**Published:** 2022-10-19

**Authors:** Athanasios Gkekas, Benoit Varenne, Nicole Stauf, Habib Benzian, Stefan Listl

**Affiliations:** 1 Department of Health Sciences, York Trials Unit, University of York, York, The United Kingdom; 2 NCD Department, World Health Organization, Oral Health Programme, Geneva, Switzerland; 3 London School of Hygiene and Tropical Medicine, London, United Kingdom; 4 Department of Epidemiology & Health Promotion, WHO Collaborating Centre Quality Improvement and Evidence-based Dentistry, College of Dentistry, New York University, New York, New York, United States of America; 5 Department of Dentistry—Quality and Safety of Oral Healthcare, Radboud University Medical Center, Radboud Institute for Health Sciences, Nijmegen, The Netherlands; Teesside University, UNITED KINGDOM

## Abstract

**Background:**

Fluoride toothpaste (FT) has recently been included in the WHO Model List of Essential Medicines. Whereas it is essential for preventing dental caries, its current affordability around the globe remains unclear. This study aimed to analyse the affordability of FT in as many as possible countries worldwide, to capture the extent of variations in FT affordability between high-, middle- and low-income countries.

**Methods:**

A standardized protocol was developed to collect country-specific information about the characteristics of the cheapest available FT at a regular point of purchase. 82 members of the WHO Global Oral Health Network of Chief Dental Officers (CDOs), directors of WHO Collaborative Centres and other oral health experts collected data using mobile phone technology. In line with established methodologies to assess affordability, the Fluoride Toothpaste Affordability Ratio (FTAR) was calculated as the expenditure associated with the recommended annual consumption of FT relative to the daily wage of the lowest-paid unskilled government worker (FTAR >1 = unaffordable spending on fluoride toothpaste).

**Results:**

There are significant differences in the affordability of FT across 78 countries. FT was strongly affordable in high-income countries, relatively affordable in upper middle-income countries, and strongly unaffordable in lower middle-income and low-income countries. The affordability of FT across WHO Regions was dependent upon the economic mix of WHO Regions’ member states.

**Conclusion:**

FT is still unaffordable for many people, particularly in low-income settings. Strategies to improve the universal affordability of FT should be part of health policy decisions in order to contribute to reducing dental caries as a global public health problem.

## Introduction

Dental caries is among the most prevalent non-communicable diseases [1,2]. The 2017 Global Burden of Disease (GBD) study estimates 20.7 age-standardised healthy years per 100,000 people are foregone due to caries of permanent teeth, indicating that dental caries affects the quality of life for many individuals [1,2]. High consumption of free sugars is a key risk factor for dental caries [3], but poor oral hygiene and limited exposure to appropriate levels of fluoride as protective factors also contribute to the high global disease burden [4].

Dental diseases, including dental caries, have a major economic impact on both individuals and healthcare systems [5,6], while most countries have little or no public coverage for oral healthcare [7]. In low- and middle-income countries (LMICs), out-of-pocket oral healthcare payments are among the common reasons for catastrophic expenditures for households [8]. The World Health Survey revealed inequitable oral healthcare coverage across 52 countries, with inequities being stronger in low-income countries [9]. Therefore, universal population-wide preventive strategies are recommended so that the burden of oral diseases is reduced and the likelihood of catastrophic expenditures related to dental treatment lowered.

The World Health Organization (WHO) promotes the use of fluorides as a key preventive strategy for dental caries [10], combined with measures to reduce the consumption of free sugars [6]. Fluoride toothpaste (FT) is the main source of topical fluoride [11,12], and an essential pillar in the prevention and control of caries, especially when alternative sources of fluoride, such as water fluoridation, are not available. There is good evidence that fluoride concentrations of 1000 and 1500 ppm in toothpaste are effective in reducing the incidence and prevalence of dental caries [11,12]. The WHO has recently added FT to its model list of essential medicines [13]. FT is a generally regulated as a cosmetic product with fluoride concentrations of up to 1500ppm, and as medical product subject to prescriptions with higher concentrations of up to 5000ppm [14]. Data on current use of FT is not readily available, even production and global sales data from manufacturers is not generally and publicly available. Surveys capturing population toothbrushing behaviour often do not ask about the use of FT for toothbrushing so that comparable global information is not available. The FDI World Dental Federation conservatively estimated in 2015 that 1.5 billion individuals worldwide use FT on a daily basis [15].

Fluoride toothpaste as a hygiene commodity and caries-protective product has not received the public policy attention that other essential medicines have enjoyed. Indications are, that due to a lack of quality control and government oversight, FT in low-income countries may contain insufficient levels of effective fluoride [14,16]. Public promotion of using FT regularly has been patchy, particularly in poorer countries [17]. Most importantly, FT may not be universally affordable, especially for poor and disadvantaged population groups who suffer from a higher burden of oral disease. The only previous study on FT affordability confirmed that cost is a barrier to universal compliance with the recommendation of brushing teeth twice daily with a FT. It is imperative that FT with fluoride concentrations of between 1000-1500ppm [13], apart from being universally available, be universally affordable according to WHO guidance for essential medicines [18].

There is no commonly agreed definition of the affordability of FT. We define such affordability as the household’s ability to pay for FT, in relation to its economic resources available for consumption [19,20]. Using the WHO and Health Action International (HAI) approach of expressing affordability in relation to days of work of the lowest-paid unskilled government worker category [21], the only published study of FT affordability in 48 countries found significant variations, with lower- and middle-income countries facing the highest burden [22]. We undertook a larger study covering more countries to analyse FT affordability, using a similar methodology and a new dimension to capture a more realistic picture to estimate FT affordability and accessibility of FT products.

## Methods

### Derivation and computation of the Fluoride Toothpaste Affordability Ratio (FTAR)

The WHO/HAI approach uses the lowest-paid unskilled government worker’s daily wage as the threshold for a medical product treating an acute condition to be affordable. If more than one day of work is required to buy a medical product, it becomes unaffordable [21].

We developed the Fluoride Toothpaste Affordability Ratio (FTAR) to assess FT affordability. Assuming 182.5g is the recommended annual consumption of FT [23], it is an unaffordable medical product in a given country when FTAR is greater than 1 calculated with Eq 1:

$$FTAR=\frac{price\;per\;g\;of\;fluoride\;toothpaste*182.5}{daily\;wage\;of\;the\;lowest-paid\;unskilled\;government\;worker}$$ (1)

If FTAR≤1, purchasing FT is affordable. We assume the poorest individuals to preferentially buy the cheapest available FT in terms of price/gram.

Moreover, consumer purchasing decisions of FT are also influenced by availability, marketing and individual preferences. Therefore, data from the Euromonitor database^19^ providing information about the three FT brands with the largest market shares for 52 countries, 40 of which were included in our study, were considered. In this case, the FTAR was modified, and FT became an unaffordable medical product in a given country when the FTAR was greater than 1 calculated with Eq 2:

$$FTAR=\frac{price\;per\;g\;of\;the\;cheapest\;top\;3\;selling\;fluoride\;toothpaste*182.5}{daily\;wage\;of\;the\;lowest-paid\;unskilled\;government\;worker}$$ (2)

Eq (1) has been applied for countries, for which data on the top-three selling FTs was unavailable from Euromonitor [24]. The FTAR for countries, for which data was available from Euromonitor, was determined according to their cheapest top-three selling FT brand, i.e., not the cheapest FT brand overall necessarily. Therefore, we considered both Eqs (1) and (2) for computing FTARs across countries.

### Study design

To obtain information related to the FTAR nominator (Eq (1) for non-Euromonitor countries or Eq (2) for Euromonitor countries), a standardised protocol was developed by the WHO Oral Health Programme Office in English, Spanish and French. The protocol asked the respondents to submit pictures through mobile phones, presenting information about the ingredients, price, package size, and full product name of FT products in their country of residence (*S1 File*). The invited respondents were either members of the WHO Global Oral Health Network of Chief Network Dental Officers (CDO), directors of WHO Collaborative Centres, or other oral health experts, and received invitation to the study via e-mail from the WHO Global Oral Health Programme. The respondents’ pictures were received by e-mail, or a mobile messaging app, and then were saved, compiled, and processed in Microsoft Excel, to estimate the FTARs. As our study’s findings are part of the upcoming WHO Global Oral Health Report, the invited respondents were informed that their submission of pictures would contribute to this report.

The protocol targeted the cheapest available product at a common point of purchase, selected of the country-specific top-three selling FT products according to Euromonitor [24]. The point of purchase was defined as a place where families or households would usually buy their supplies. It was up to the responders’ judgment to determine the most relevant common point of purchase in their country of residence. When the top-three selling FT brands could not be identified for a specific country, the data collection targeted the cheapest FT available at a common point of purchase instead. The sample protocol for Euromonitor countries is shown in *S1 File*. The sample protocol for non-Euromonitor countries is shown in *S2 File*.

The main aim of such a design was to collect FT data from as many LMICs as possible, and from all WHO Regions, in the most convenient way possible for the invited responders. To ensure homogeneity in the computation of FTAR figures, we reported the package size of all FTs in grams; therefore, if there was no package information regarding the density of g/ml in a FT, the package size of which was originally reported in ml, we assumed a density of 1.30 g/ml to convert the figure. The data collection took place between June 2019 and September 2019.

### Data entry

With respect to the FTAR denominator, we estimated the “daily wage of the lowest-paid unskilled government worker” through proxies. For most (71 of 78) sampled countries, the daily expenditures per capita of the poorest 15% of the population was the corresponding proxy. For 64 countries, we computed such a proxy by using the Households and Non-Profit Institutions Serving Households (NPISHs) Final consumption expenditure in constant USD PPP rates, the most recent population estimates, and the averages of the most recently reported shares of national income held by the poorest 10% and poorest 20% of the population [25–28]. The most recent estimates of these indicators were available for the year 2017 at the time of data entry. For seven countries (Solomon Islands, Saint Kitts and Nevis, Lesotho, Myanmar and Fiji), we computed the proxy by using the Gross National Income (GNI) in constant USD PPP rates [29], as the NPISHs Final Consumption expenditure was unavailable, the most recent population estimates and the averages of the shares of national income held by the poorest 10% and 20% of the population. We applied the GDP deflator to two of these countries (Myanmar and Solomon Islands) as their latest available GNI data were before 2017 [30].

We used the daily minimum wage, as of 2019, as an alternative proxy for seven (of 78) sampled countries (Trinidad and Tobago, Saint Kitts and Nevis, Cambodia, Central African Republic, Japan, Hong Kong SAR (China) and New Zealand), for which no national income share data were available [31]. Minimum wages reported in months (years) were converted to daily minimum wages by dividing monthly (annual) minimum wages by 30 (365). If minimum wages were reported in hours, we assumed an average working time of eight hours per day to obtain the daily figures.

With respect to the FTAR nominator, the obtained 2019 prices/g of the cheapest (top-three selling) FTs in local currency units were converted to the corresponding USD PPP rates [32], and then were adjusted to 2017 prices/g in USD PPP rates through the GDP deflator, to match the 2017 figures [30], which were the most recent available estimates for use in the computation of FTARs at the time of data analysis. When the daily minimum wage was the alternative proxy, we made no inflation adjustments, as the figures corresponded to year 2019.

### Estimation of FTARs and statistical analysis

We computed the FTARs of all countries according to the extracted primary data regarding the price/g of the cheapest (top-three selling) FT, related to the nominator of the FTAR, and the estimates of the daily wage of the lowest-paid unskilled government worker, related to the denominator of the FTAR. We also estimated and discussed the corresponding FTAR descriptive statistics. Additionally, we estimated the variations in FTAR figures by different World Bank Income Groups i.e., high-income group, upper middle-income group, lower middle-income group and low-income group, through stratified descriptive statistics, to explore the potential variations in the affordability of FT around the globe. Furthermore, we estimated the variations in FTAR figures by different WHO Regions.

To confirm whether the variations in the affordability of FT across Income Groups (and WHO Regions) existed because of potential FT price variations, i.e., variations in the nominator of the FTAR, or solely due to variations in the daily wages of the lowest-paid unskilled government worker, i.e., variations in the denominator of the FTAR, we also undertook two-sample-t-tests of the difference in mean prices/g of the cheapest (top-three selling) FTs between all pairs of World Bank Income Groups and WHO Regions.

## Results

Using the responses to the protocol from 82 participants, 60% of whom were members of the WHO Global Oral Health Network, we analysed primary data of FT products from 78 countries (40 Euromonitor and 38 non-Euromonitor countries). Data from four Euromonitor countries (Greece, Hong Kong, Switzerland, United Kingdom) were collected by two independent participants. In all four countries the collected information on the top-three selling FT products was identical among all respondents.

*Table 1* provides the number and proportions of countries included in the data analysis by Euromonitor, WHO Regions and income groups. 56% of the sample consists of LMICs, whereas 44% consists of high-income countries. Data related to the affordability of FT were compiled for nearly 40% of all WHO member states.

**Table 1:** Countries included in the study by Euromonitor, WHO Region and Income Group.

**Euromonitor status**	**Number of countries and administrative regions**	**Proportion in terms of total countries included in the study (%)**			
Data available from Euromonitor	40	51.28			
Data unavailable from Euromonitor	38	48.72			
Total	78	100.00			
**WHO Region**	**Number of countries**	**Proportion of total countries included in the study (%)**		**Proportion of total countries in the WHO Region**	
EURO	30 (of 53)	38.96		56.60	
AFRO	18 (of 47)	23.38		38.30	
WPRO	11 (of 27)	14.29		40.74	
PAHO	10 (of 35)	12.99		28.57	
SEARO	5 (of 11)	6.49		45.45	
EMRO	3 (of 22)	3.89		13.64	
Total	77 (of 195)	100.00		39.69 (Of all WHO Member States)	
**World Bank Income Group**	**Number of countries and administrative regions**	**Proportion in terms of total countries considered in the study (%)**		**Proportion in terms of total countries in the World Bank Income Group**	
High-income	34(of 80)	43.59		42.50	
Upper middle-income	19(of 55)	24.36		34.55	
Lower middle-income	18(of 55)	23.08		32.73	
Low-income	7(of 27)	8.97		25.93	
Total	78 (of 217)	100.00		35.94	
**WHO Region**	**High-income countries (%)**	**Upper middle-income countries (%)**	**Lower middle-income countries (%)**	**Low-income countries (%)**	**Total**
EURO	25 (83.33%)	5 (16.67%)	0	0	30
AFRO	0	2 (11.11%)	9 (50.00%)	7 (38.89%)	18
PAHO	4 (40.00%)	6 (40.00%)	0	0	10
WPRO	5* (41.67%)	3 (25.00%)	4 (33.33%)	0	11 *
EMRO	0	2 (66.67%)	1 (33.33%)	0	3
SEARO	0	1 (20.00%)	4 (80.00%)	0	5
Total	34 (43.58%)	19 (24.36%)	18 (23.07%)	7 (9.00%)	**78**

* Note: Hong Kong SAR is an Administrative Region of the People’s Republic of China, and not a distinct WHO member state. However, the Euromonitor data are different for Hong Kong and the People’s Republic of China. Therefore, the total number of countries and administrative regions by Euromonitor status and World Bank is 78 and the total number of countries by WHO Region is 77. Since Hong Kong SAR lies in the WPRO Region, as an administrative region of the People’s Republic of China, its data are considered separately from People’s Republic of China when estimating the FTAR descriptive statistics of the WPRO Region.

**Fig 1:**
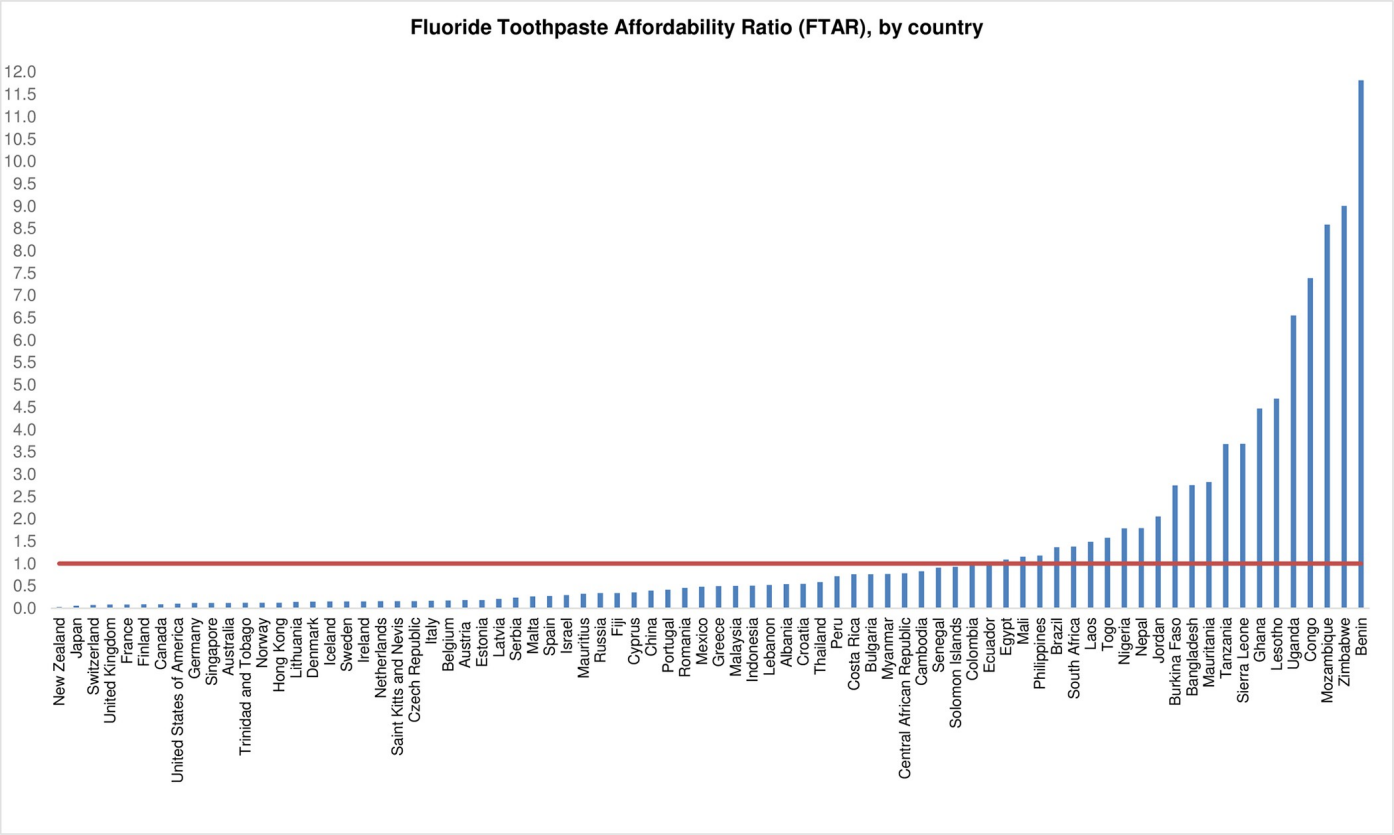
Fluoride Toothpaste Affordability Ratio (FTAR), by country.

FTARs and prices/g of the cheapest (top-three selling) FT from each included country in the sample are displayed in *Table 2*, in ascending order in terms of FTARs. The green faded countries did not experience any unaffordable expenditures on the annual recommended amount of FT, whereas the red faded countries experienced unaffordable expenditures on the annual recommended amount of FT. A graphical representation of FTARs by country, along with the FTAR = 1 affordability threshold (shown in a red horizontal line), is provided in *Fig 1*.

**Table 2:** Fluoride Toothpaste Affordability Ratio (FTAR), and price per gram of the cheapest (top-three selling) FT, by country.

Included in Euromonitor (Database indicating top-three selling FT)	Country		WHO Region	World Bank Income Group	Fluoride Toothpaste Affordability Ratio (FTAR)/ Working days needed to buy the cheapest (top three selling) FT	Price per gram of the cheapest top-three selling fluoride toothpaste (USD PPP rates, 2011)
No		Albania	EURO	Upper-middle	0.5398	0.024
Yes		Australia	WPRO	High	0.1206	0.013
Yes		Austria	EURO	High	0.1851	0.023
No		Bangladesh	SEARO	Lower middle	2.7546	0.039
Yes		Belgium	EURO	High	0.1728	0.022
No		Benin	AFRO	Lower middle	11.8159	0.032
Yes		Brazil	PAHO	Upper-middle	1.3673	0.023
Yes		Bulgaria	EURO	Upper-middle	0.7650	0.031
No		Burkina Faso	AFRO	Low	2.7483	0.015
No		Cambodia	WPRO	Lower middle	0.8276	0.027
Yes		Canada	PAHO	High	0.0899	0.010
No		Central African Republic	AFRO	Low	0.7821	0.020
Yes		China	WPRO	Upper-middle	0.3951	0.010
Yes		Colombia	PAHO	Upper-middle	0.9592	0.022
No		Congo	AFRO	Lower middle	7.3898	0.031
No		Costa Rica	PAHO	Upper-middle	0.7631	0.024
No		Croatia	EURO	High	0.5438	0.031
No		Cyprus	EURO	High	0.3557	0.029
Yes		Czech Republic	EURO	High	0.1608	0.016
Yes		Denmark	EURO	High	0.1506	0.020
No		Ecuador	PAHO	Upper-middle	0.9902	0.018
Yes		Egypt	EMRO	Lower middle	1.0906	0.055
No		Estonia	EURO	High	0.1857	0.013
No		Fiji	WPRO	Upper-middle	0.3413	0.016
Yes		Finland	EURO	High	0.0883	0.012
Yes		France	EURO	High	0.0846	0.009
Yes		Germany	EURO	High	0.1181	0.015
No		Ghana	AFRO	Lower middle	4.4733	0.045
Yes		Greece	EURO	High	0.4938	0.030
Yes		Hong Kong	WPRO	High	0.1230	0.018
No		Iceland	EURO	High	0.1524	0.025
Yes		Indonesia	SEARO	Lower middle	0.5074	0.013
Yes		Ireland	EURO	High	0.1557	0.017
Yes		Israel	EURO	High	0.2964	0.019
Yes		Italy	EURO	High	0.1704	0.013
Yes		Japan	WPRO	High	0.0582	0.011
No		Jordan	EMRO	Upper-middle	2.0536	0.076
No		Laos	WPRO	Lower middle	1.4869	0.027
No		Latvia	EURO	High	0.2104	0.013
No		Lebanon	EMRO	Upper-middle	0.5217	0.033
No		Lesotho	AFRO	Lower middle	4.6939	0.022
No		Lithuania	EURO	High	0.1459	0.010
Yes		Malaysia	WPRO	Upper-middle	0.5008	0.027
No		Mali	AFRO	Low	1.1564	0.009
No		Malta	EURO	High	0.2644	0.024
No		Mauritania	AFRO	Lower middle	2.8241	0.026
No		Mauritius	AFRO	Upper-middle	0.3249	0.021
Yes		Mexico	PAHO	Upper-middle	0.4794	0.019
No		Mozambique	AFRO	Low	8.5889	0.023
No		Myanmar	SEARO	Lower middle	0.7694	0.021
No		Nepal	SEARO	Lower middle	1.7960	0.020
Yes		Netherlands	EURO	High	0.1575	0.020
Yes		New Zealand	WPRO	High	0.0279	0.011
Yes		Nigeria	AFRO	Lower middle	1.7870	0.021
Yes		Norway	EURO	High	0.1228	0.019
Yes		Peru	PAHO	Upper-middle	0.7178	0.017
Yes		Philippines	WPRO	Lower middle	1.1778	0.028
Yes		Portugal	EURO	High	0.4158	0.031
Yes		Romania	EURO	Upper-middle	0.4564	0.020
Yes		Russia	EURO	Upper-middle	0.3377	0.022
No		Saint Kitts and Nevis	PAHO	High	0.1605	0.036
No		Senegal	AFRO	Lower middle	0.9108	0.012
No		Serbia	EURO	Upper-middle	0.2407	0.013
No		Sierra Leone	AFRO	Low	3.6790	0.025
Yes		Singapore	WPRO	High	0.1205	0.024
No		Solomon Islands	WPRO	Lower middle	0.9302	0.007
Yes		South Africa	AFRO	Upper-middle	1.3809	0.016
Yes		Spain	EURO	High	0.2766	0.019
Yes		Sweden	EURO	High	0.1553	0.019
Yes		Switzerland	EURO	High	0.0752	0.012
No		Tanzania	AFRO	Lower middle	3.6763	0.026
Yes		Thailand	SEARO	Upper-middle	0.5885	0.024
No		Togo	AFRO	Low	1.5773	0.009
No		Trinidad and Tobago	PAHO	High	0.1218	0.018
No		Uganda	AFRO	Low	6.5530	0.032
Yes		United Kingdom	EURO	High	0.0843	0.011
Yes		United States of America	PAHO	High	0.1029	0.013
No		Zimbabwe	AFRO	Lower middle	9.0038	0.074

From the sampled countries, an average lowest-paid unskilled government worker needed more than one day of work to purchase 182.5g of the cheapest (top-three selling) FT, a figure above the affordability threshold (FTAR = 1) (*Table 3*). The lower bound of the FTAR 95% confidence interval was below the threshold, whereas the upper bound was above the threshold, reflecting FT affordability variations across the sampled countries. 22 of 78 countries showed FTARs that would result in unaffordable expenditures on purchasing the annual recommended amount of the cheapest (top-three selling) FT (*Table 2*, *Fig 1*).

**Table 3:** Descriptive statistics for full samples, in FTARs.

	Fluoride Toothpaste Affordability Ratio (FTAR)
**Mean (95% Confidence Interval)**	1.32 (95% CI 0.82 to 1.81)
**Standard Deviation**	2.2294
**Maximum**	11.8159
**Minimum**	0.0279
**Median**	0.4866
**Number of countries (= n)**	78
**Number of WHO Member states (= n)** *	77

* Including Hong Kong, China SAR (Special Administrative Region of People’s Republic of China).

The sampled high-income countries had the lowest mean FTAR among the four income groups, with an average lowest-paid unskilled government worker needing only 0.18 (95% CI 0.14 to 0.22) working days to purchase 182.5g of the cheapest (top-three selling) FT (*Table 4*). New Zealand, a high-income country, had the lowest reported FTAR (= 0.0279) of the 78 sampled countries, whereas Croatia had the highest reported FTAR (= 0.5438) across high-income countries, but still below the affordability threshold of FTAR = 1. No high-income country in the sample experienced unaffordable expenditures on 182.5g of the cheapest (top-three selling) FT.

**Table 4:** Descriptive Statistics by World Bank Income Groups, in FTARs.

FTAR by Income Group	High-income countries	Upper middle-income countries	Lower middle-income countries	Low-income countries
**Mean (95% Confidence Interval)**	0.18 (95% CI 0.14 to 0.22)	0.72 (95% CI 0.52 to 0.93)	3.22 (95% CI 1.74 to 4.70)	3.58 (95% CI 1.39 to 5.77)
**Standard Deviation**	0.1186	0.4603	3.2039	2.9536
**Maximum**	0.5438	2.0536	11.8159	8.5889
**Minimum**	0.0279	0.2407	0.5074	0.7821
**Median**	0.1539	0.5398	1.7915	2.7483
**Number of countries (= n)**	34*	19	18	7
**Number of WHO Member states (= n)**	33	19	18	7
**Percentage of included countries experiencing unaffordable expenditures on FT**	0%	15.79%	72.22%	85.71%

* Including Hong Kong, China SAR (Special Administrative Region of People’s Republic of China).

In the sampled upper middle-income countries, an average lowest-paid unskilled government worker needed 0.72 (95% CI 0.52 to 0.93) working days to purchase 182.5g of the cheapest (top-three selling) FT (*Table 4*). Although this number is below the threshold for unaffordable expenditure, strong variations existed. For instance, in Jordan (FTAR = 2.0536) it was unaffordable to buy 182.5g of FT. 16% of the sampled upper middle-income countries experienced unaffordable expenditures on 182.5g of the cheapest (top-three selling) FT.

In the sampled lower middle-income countries, an average lowest-paid unskilled government worker needed 3.22 (95% CI 1.74 to 4.70) working days to purchase 182.5g of the cheapest (top-three selling) FT (*Table 4*). Benin, a lower middle-income country, had the highest reported FTAR (= 11.8159) of the 78 sampled countries. 72% of the sampled lower middle-income countries experienced unaffordable expenditures on 182.5g of the cheapest (top-three selling) FT.

In the sampled low-income countries, an average lowest-paid unskilled government worker needed 3.58 (95% CI 1.39 to 5.77) working days to purchase 182.5g of the cheapest (top-three selling) FT (*Table 4*). The highest unaffordable expenditure was observed in Mozambique (FTAR = 8.5889). Most low-income countries (86% in the sample) experienced unaffordable expenditures on 182.5g of the cheapest (top-three selling) FT, with the only exception being the Central African Republic (FTAR = 0.7821).

A corresponding FTAR analysis according to WHO Regions *(S1 Table)* was undertaken. The findings depended upon the geographical clustering of the sampled countries though affordability is not related to geography, but to a country’s economic situation. Unaffordable expenditures on fluoride toothpaste were mainly observed in the African Region (AFRO), and in some countries in the remaining WHO regions but the European Region (EURO).

There were no significant FT price/g variations among the income groups, according to two-sample t-tests, which considered all possible pairs of income groups (*S2 Table).* The only exception was the FT price comparison in high-income countries versus lower middle-income countries, with high-income countries experiencing a mean price/g of the cheapest (top-three selling) FT costing $0.01 less than the mean price/g of FT in lower middle-income countries. Similar to the analysis according to income groups, there were no significant FT mean price/g variations among WHO Regions.

## Discussion

### Implications of the findings

Our study aimed to evaluate the affordability of FT in as many countries as possible to capture the extent of variations in FT affordability between high-, middle- and low-income countries. Substantial differences in the affordability of FT among World Bank income groups were shown. From the perspective of the lowest-paid unskilled government worker, the annual recommended amount of the cheapest (top-three selling) FT would still be affordable for those living in high-income countries, in contrast to those in low-income countries. The picture in middle-income countries was more mixed, with FT being affordable in most upper middle-income countries, but not all (Brazil, Jordan and South Africa), and unaffordable in most lower middle-income countries. With respect to the analysis by WHO Regions, the variations in FT affordability were largely determined by the economic mix of the WHO Regions’ member states. If faced with unaffordable purchasing choices, consumers living on a low income may avoid buying FT products and hence miss out on their preventive benefits for oral health. Such findings add another aspect on inequalities in oral health and opportunities for effective self-care.

Interestingly, the earnings of the lowest-paid unskilled government worker, rather than variations in the prices per g of the cheapest (top-three selling) FTs, influenced the variability in FT affordability across countries in the sample. It can thus be concluded that current FT prices for populations in low-resource settings are too high related to their earnings. Recent investment case estimates for Burkina Faso, a low-income country, indicate that FT prices would need to be 33% lower than current market prices for a consumer to invest in oral health [33].

### Study strengths and weaknesses

Compared to the previous study [22], our study provides evidence on FT affordability from a higher number of countries, covering approximately 40% of WHO member states and including a considerable number of LMICs (44). Moreover, the study’s design reduces the risk of reporting errors in the collection of the FT pricing data through the submission of participant responses via photos instead of self-completed questionnaires. This also increases the overall response rate since only a minimal amount of time during normal shopping is required to collect a response. Furthermore, our study has introduced a new dimension for estimating the FTAR across countries, that is the consideration of the availability, individual preferences, and marketing of FTs at the national level, by asking participants from 40 of 78 included countries to provide pricing information about the cheapest top-three selling FT (according to Euromonitor database [24]) in their country of residence. Finally, we consider the FTAR as a reliable measure for international comparisons of FT affordability, because it is aligned with to the widely adopted WHO/HAI approach [21].

However, four limitations exist. First, we made several approximations of the daily wage of the lowest-paid unskilled government worker to obtain the FTAR figures. For instance, the estimated FTARs of seven countries, which used daily minimum wages as a proxy for the daily wage of the lowest-paid unskilled government worker, may be different from the corresponding FTARs that would have been estimated had the daily expenditure per capita of the poorest 15% of the population data been available with appropriate income shares. Second, consumers do not tend to buy the full annual supply of FT at once; they usually buy it in smaller increments, either when a tube or sachet is empty, or when there is a special bargain at a common point of purchase. Therefore, the theoretical construct of unaffordable expenditure may not match the actual FT consumption behaviour of many individuals. Nevertheless, as this limitation was identified before the data collection process, the study’s protocol asked the responders to provide information about FTs with a package size ranging as closely as possible to 75–100 ml/g. Third, the definition of FTAR may not accurately represent the consumption behaviour of poorer economic groups. Several dependents may rely financially upon the single earning of a lowest-paid unskilled government worker, thus making the earning indirectly lower for such a worker [21]. Fourth, it is likely that regional variations in FTAR within the surveyed countries exist, either due to variations in the income of the lowest-skilled government worker or variations in the cost of the cheapest (top-3 selling) FT product; such variations were not captured in our study.

### Policy recommendations for strengthening the universal affordability of FT

The study has shown that the affordability of FT needs to be addressed as a matter of public health concern at least in countries with a risk of unaffordable expenditure, mainly LMICs. Entry points for interventions, leading to lower consumer prices and better affordability, are related to government regulations that influence the prices of medical commodities, such as FT. For other medicines and medical products, equity pricing has been used, where prices are differentiated according to the country’s or population group’s income levels or purchasing power.

The World Trade Organization (WTO) suggests strategies such as generic competition, high volume production “through global or regional procurement”, the adoption of differential pricing, or the strengthening of local production “through voluntary licensing and technology transfer” as viable approaches to make essential medicines more affordable [34]. Similar strategies might be applied for FT as well, ideally aligned with national policies related to essential medicines. In controlled contexts of community, school or workplace programmes policies should be encouraged that provide FT free of charge if financially feasible, to improve the oral health outcomes of people living in socioeconomically deprived areas [35].

Fiscal measures may include the elimination or reduction of VAT for essential medicines or medical products to strengthen consumer demand [36]. Any reduction in VAT or other tariffs would be beneficial for the FT due to the regressive nature of such levies affecting poorer households more than richer households. It is important, however, that any reductions in taxes and tariffs are also passed on to the consumer rather than being used to increase the producer or distributor’s profit margins.

Finally, profit margins for toothpaste products along the production and logistics chains are significant. Packaging and marketing have a large share in production costs [37], which leads to a low-price segment of unbranded products sold by large retailers. Promoting generic competition through social marketing strategy for price reduction, however, is complex and requires a solid framework of quality assurance and standards for FT. Product quality, labelling requirements and consumer safety are matters of public health concern, including a large market share of counterfeit products in the low-price segments in many countries [38].

The WHO Director-General’s report on oral health recommends “promoting legislation to increase the affordability and accessibility of high-quality fluoride toothpaste and advocating for its recognition as an essential health product” [39]. The recent inclusion of FT in the WHO Model List of Essential Medicines [13], as well as our findings, provide a unique and pertinent opportunity to address FT affordability depending on national fiscal setting, taxation systems and regulations, and supported by additional public policy actions to ensure universal access FT.

### Conclusions

Fluoride toothpaste (FT) is an essential public health product to reducing the incidence of dental caries, one of the most prevalent non-communicable diseases worldwide. Despite its wide availability for citizens across the globe, FT’s potential benefits are not fully achieved due to high prices for the poor and disadvantaged populations in many middle- and low-income countries. To ensure that everyone has access to FT, health policy options should be considered to address and improve the universal affordability of FTs, such as equity pricing, generic competition, encouragement of local production of FTs, and proper elimination or reduction of VAT for FTs.

## Supporting information

S1 Fig Standardised protocol for Euromonitor countries. (TIF)

S2 Fig Standardised protocol for non- Euromonitor countries. (TIF)

S1 Table Descriptive statistics by WHO Regions, in FTARs. (DOCX)

S2 Table Results from two-sample-t-tests of the difference in mean prices/g of the cheapest (top-three selling) FTs between all pairs of World Bank Income Groups and WHO Regions. (DOCX)

S1 File Standardised protocol for Euromonitor countries. (DOCX)

S2 File Sample protocol for non-Euromonitor countries. (DOCX)

S3 File FTAR analysis by WHO Region. (DOCX)

S4 File Differences in mean price/g of the cheapest (top-three selling) FTs among World Bank Income Groups and WHO Regions. (DOCX)
