# Supplementary figures and images for: Affordability of essential medicines: The case of fluoride toothpaste in 78 countries
Source: PLoS One. 2022 Oct 19;17(10):e0275111. doi: 10.1371/journal.pone.0275111 (PMC9581416; doi:10.1371/journal.pone.0275111)

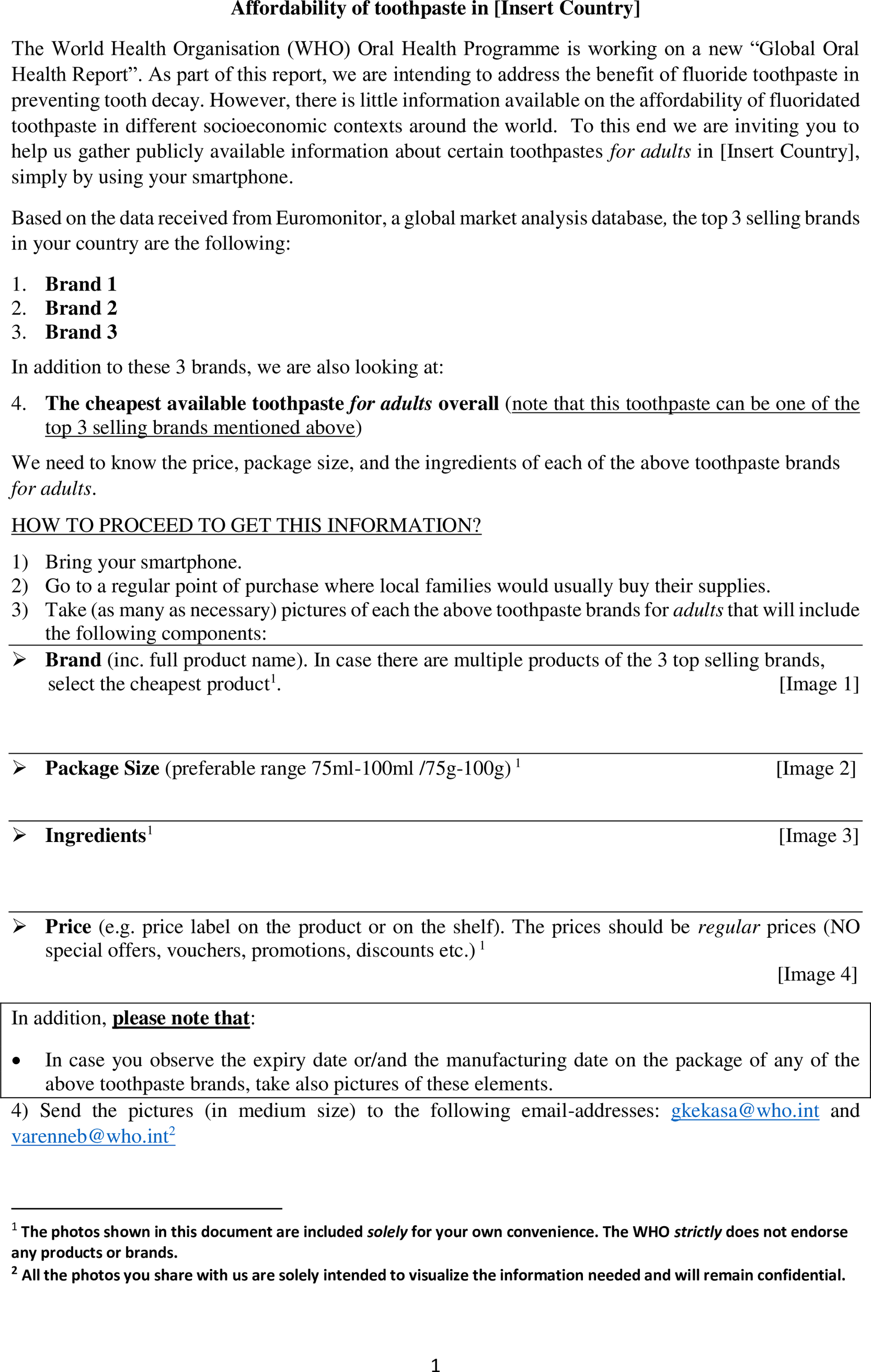

Supplement: S1 Fig — (TIF) [file pone.0275111.s001.tif]

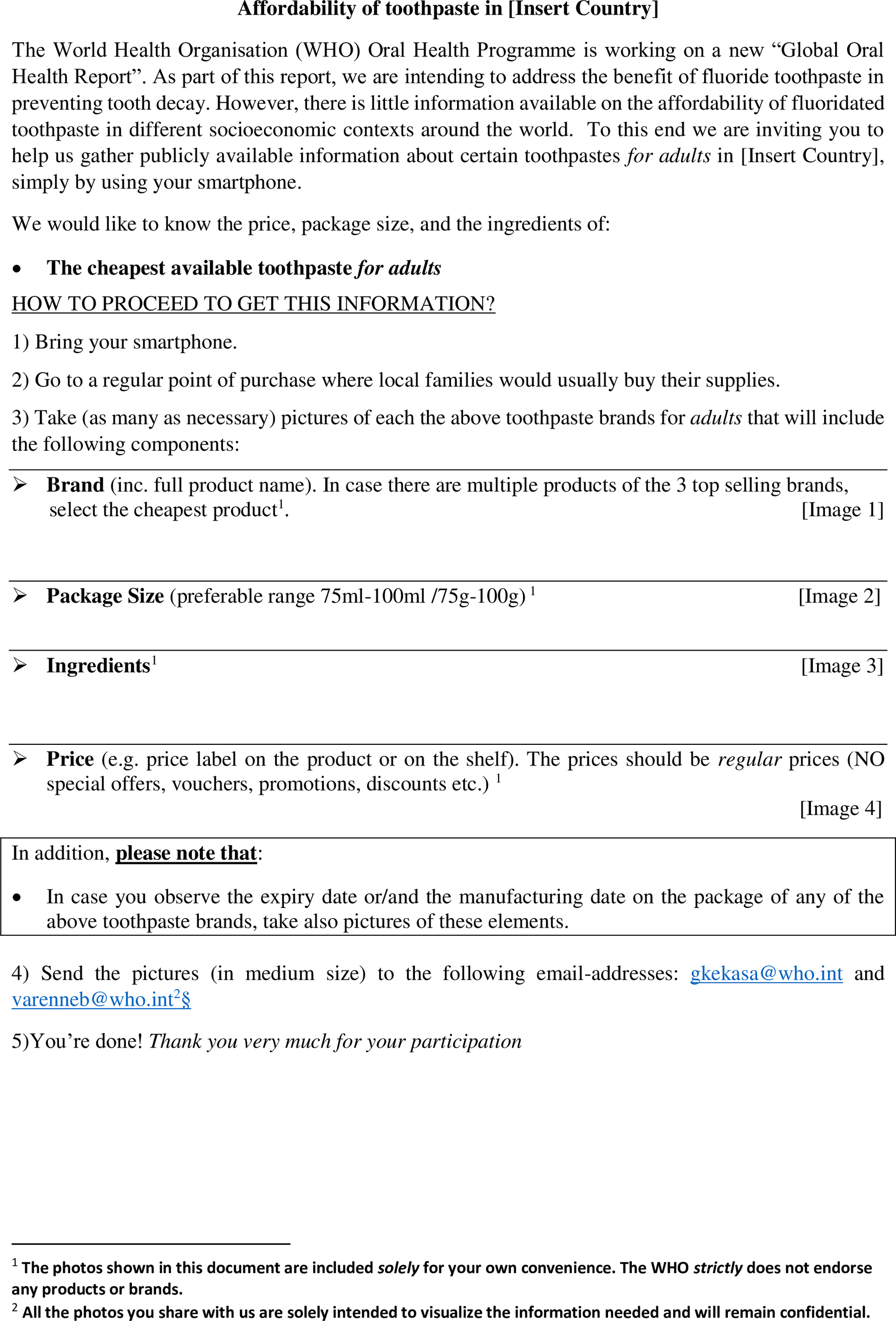

Supplement: S2 Fig — (TIF) [file pone.0275111.s002.tif]
