# Supplementary material for: Affordability of essential medicines: The case of fluoride toothpaste in 78 countries
Source: PLoS One. 2022 Oct 19;17(10):e0275111. doi: 10.1371/journal.pone.0275111 (PMC9581416; doi:10.1371/journal.pone.0275111)
Supplement: S1 Table — (DOCX) [file pone.0275111.s003.docx]

| **FTAR by WHO Region** | **EURO** | **AFRO** | **PAHO** | **WPRO** | **EMRO** | **SEARO** |
| --- | --- | --- | --- | --- | --- | --- |
| **Mean (95% Confidence Interval)** | 0.25 (95% CI 0.19 to 0.31) | 4.08 (95% CI 2.54 to 5.62) | 0.58 (95% CI 0.29 to 0.86) | 0.51 (95% CI 0.21 to 0.81) | 1.22 (95% CI 0.35-2.10) | 1.28 (95% CI 0.43 to 2.14) |
| **Standard Deviation** | 0.1680 | 3.3061 | 0.4531 | 0.4870 | 0.7744 | 0.9717 |
| **Maximum** | 0.7650 | 11.8159 | 1.3673 | 1.4869 | 2.0536 | 2.7546 |
| **Minimum** | 0.0752 | 0.3249 | 0.0899 | 0.0279 | 0.5217 | 0.5074 |
| **Median** | 0.17895 | 3.2502 | 0.5986 | 0.3682 | 1.0906 | 0.7694 |
| **Number of countries (=n)** | 30 | 18 | 10 | 12* | 3 | 5 |
| **Number of WHO Member states (=n)** | 30 | 18 | 10 | 11 | 3 | 5 |
| **Percentage of included countries experiencing**  **unaffordable expenditure on FT** | 0% | 83.33% | 10% | 16.66% | 66.67% | 40% |

* Including Hong Kong, China SAR (Special Administrative Region of People’s Republic of China)
