# Supplementary material for: Affordability of essential medicines: The case of fluoride toothpaste in 78 countries
Source: PLoS One. 2022 Oct 19;17(10):e0275111. doi: 10.1371/journal.pone.0275111 (PMC9581416; doi:10.1371/journal.pone.0275111)
Supplement: S2 Table — (DOCX) [file pone.0275111.s004.docx]

| Combination of World Bank Income Groups or WHO Regions (qualitative) | Combination of World Bank Income Groups or WHO Regions (in terms of i, l) | Sample difference in the average price/g of the cheapest (top-three selling) FTs  (p_1il_-p_2il_) | Pooled standard deviation  (s_il_) | Degrees of freedom  (v) | Absolute t statistic  (\| t_il_ \|) | Critical t-value (t_va_) | Conclusion |
| --- | --- | --- | --- | --- | --- | --- | --- |
| EURO Region against AFRO Region | i=1,l=0 | -0.006 | 0.0106 | 46 | 1.9206 | 2.013 | No difference in average prices |
| EURO Region against WPRO Region | i=2,l=0 | 0.001 | 0.0071 | 40 | 0.4426 | 2.021 | No difference in average prices |
| EURO Region against SEARO Region | i=3,l=0 | -0.004 | 0.0071 | 33 | 1.1694 | 2.035 | No difference in average prices |
| EURO Region against PAHO Region | i=4,l=0 | -0.001 | 0.0068 | 38 | 0.2895 | 2.024 | No difference in average prices |
| EURO Region against EMRO Region | i=5,l=0 | -0.035 | 0.0085 | 31 | 6.7948 | 2.040 | Difference in average prices |
| AFRO Region against WPRO Region | i=6,l=0 | 0.007 | 0.0128 | 28 | 1.5008 | 2.048 | No difference in average prices |
| AFRO Region against SEARO Region | i=7,l=0 | 0.002 | 0.0142 | 21 | 0.2898 | 2.080 | No difference in average prices |
| AFRO Region against PAHO Region | i=8,l=0 | 0.005 | 0.0129 | 26 | 1.0521 | 2.056 | No difference in average prices |
| AFRO Region against EMRO Region | i=9,l=0 | -0.029 | 0.0160 | 19 | 2.9071 | 2.093 | Difference in average prices |
| WPRO Region against SEARO Region | i=10,l=0 | -0.005 | 0.0083 | 15 | 1.1436 | 2.131 | No difference in average prices |
| WPRO Region against PAHO Region | i=11,l=0 | -0.002 | 0.0076 | 20 | 0.5517 | 2.086 | No difference in average prices |
| WPRO Region against EMRO Region | i=12,l=0 | -0.036 | 0.0112 | 13 | 4.9961 | 2.160 | Difference in average prices |
| SEARO Region against PAHO Region | i=13,l=0 | 0.003 | 0.0079 | 13 | 0.7599 | 2.160 | No difference in average prices |
| SEARO Region against EMRO Region | i=14,l=0 | -0.031 | 0.0147 | 6 | 2.8939 | 2.447 | Difference in average prices |
| PAHO Region against EMRO Region | i=15,l=0 | -0.034 | 0.0113 | 11 | 4.6254 | 2.201 | Difference in average prices |
| High-income countries against upper middle-income countries | i=0,l=1 | -0.006 | 0.0100 | 51 | 1.9460 | 2.008 | No difference in average prices |
| High-income countries against lower middle-income countries | i=0,l=2 | -0.011 | 0.0110 | 50 | 3.3449 | 2.009 | Difference in average prices |
| High-income countries against low-income countries | i=0,l=3 | -0.001 | 0.0074 | 39 | 0.2119 | 2.023 | No difference in prices |
| Upper middle-income countries against lower middle-income countries | i=0,l=4 | -0.005 | 0.0149 | 35 | 1.0528 | 2.030 | No difference in prices |
| Upper middle-income countries against low-income countries | i=0,l=5 | 0.005 | 0.0127 | 24 | 0.8789 | 2.064 | No difference in prices |
| Lower middle-income countries against low-income countries | i=0,l=6 | 0.010 | 0.0145 | 23 | 1.5647 | 2.069 | No difference in prices |
