# Supplementary material for: Affordability of essential medicines: The case of fluoride toothpaste in 78 countries
Source: PLoS One. 2022 Oct 19;17(10):e0275111. doi: 10.1371/journal.pone.0275111 (PMC9581416; doi:10.1371/journal.pone.0275111)
Supplement: S1 File — (DOCX) [file pone.0275111.s005.docx]

**Supplementary Material 1. Standardised protocol for Euromonitor countries**

The ingredients were needed to verify if the respondents had submitted toothpaste products containing the desirable levels of fluoride (1000–1500 ppm). The price, reported in 2019 domestic levels, was relevant for detecting the cheapest top-three selling FT in the case of Euromonitor countries, and the subsequent computation of FTAR in all countries, i.e., both Euromonitor and non-Euromonitor countries. The package size was required, so that we could compute the price/g of the cheapest (top-three selling) FT and use it in the nominator of the FTAR for each country. Finally, if a country belonged to the Euromonitor database of the top-three selling brands, the full product names were essential for cross-matching the names of the sampled FT products with the top-three selling brands according to the Euromonitor database.

The standardised protocol for Euromonitor countries is available below. The images in the protocol were removed because FT brands, neither endorsed by the WHO nor financially related to our study, were visible. These images were attached solely for instruction and demonstration purposes of the respondents.

**Fig S1. Standardised protocol for Euromonitor countries**
