# Supplementary material for: Affordability of essential medicines: The case of fluoride toothpaste in 78 countries
Source: PLoS One. 2022 Oct 19;17(10):e0275111. doi: 10.1371/journal.pone.0275111 (PMC9581416; doi:10.1371/journal.pone.0275111)
Supplement: S2 File — (DOCX) [file pone.0275111.s006.docx]

**Supplementary Material 2. Standardised protocol for non-Euromonitor countries**

**Fig S2. Standardised protocol for non-Euromonitor countries**
