# Supplementary material for: Affordability of essential medicines: The case of fluoride toothpaste in 78 countries
Source: PLoS One. 2022 Oct 19;17(10):e0275111. doi: 10.1371/journal.pone.0275111 (PMC9581416; doi:10.1371/journal.pone.0275111)
Supplement: S3 File — (DOCX) [file pone.0275111.s007.docx]

**Supplementary Material 3. FTAR Analysis by WHO Region**

The EURO Region, whose 83% of its members included in the sample were high-income countries and 17% of its members included in the sample were upper middle-income countries, experienced no unaffordable expenditures on 182.5g of the cheapest (top-three selling) FT. The lowest-paid unskilled government worker needed only 0.25 (95% CI 0.19 to 0.31) working days to purchase 182.5g of the cheapest (top-three selling) FT, on average (*S1 Table*). Bulgaria had the highest reported FTAR (=0.7650), but still below the affordability threshold of FTAR=1. As a result, no country in the sample belonging to the EURO Region experienced unaffordable expenditures on FT.

The PAHO Region, whose 40% of its members included in the sample were high-income countries and 60% of its members included in the sample were upper middle-income countries, did not experience unaffordable expenditures on the cheapest (top-three selling) FT, on average (*S1 Table)*. The lowest-paid unskilled government worker needed only 0.58 (95% CI 0.29 to 0.86) working days to purchase 182.5g of the cheapest (top-three selling) FT, on average. However, there were variations among the included countries, with Brazil facing unaffordable expenditures on purchasing FT (FTAR=1.3673). 10% of the included countries from the PAHO Region experienced unaffordable expenditures on FT.

The WPRO Region, whose 42% of its members included in the sample were high-income countries, 25% of its members included in the sample were upper middle-income countries, and 33% of its members included in the sample were lower middle-income countries, did not experience unaffordable expenditures on FT on average (*S1 Table*). The lowest-paid unskilled government worker needed only 0.51 (95% CI 0.21 to 0.81) working days to purchase 182.5g of the cheapest (top-three selling) FT, on average. However, there were variations among the included countries, with New Zealand having the lowest reported FTAR in the sample of all countries in the study (=0.0279) but with Laos and Philippines experiencing unaffordable expenditures on 182.5g of the cheapest (top-three selling) FT (FTAR=1.4869 and FTAR=1.1778, respectively). 17% of the included countries from the WPRO Region experienced unaffordable expenditures on the cheapest (top-three selling) FT.

However, the SEARO Region, which none of its members included in the sample were high-income countries, 20% of its members included in the sample were upper-middle- income countries, and 80% of its members included in the sample were lower middle-income countries, may be subject to unaffordable expenditures on FT (*S1 Table*). The lowest-paid unskilled government worker needed 1.28 (95% CI 0.43 to 2.14) days of work to purchase 182.5g of the cheapest (top-three selling) FT, on average. Furthermore, there was substantial variation among the included countries, with Indonesia having a low FTAR figure (=0.5074), but with Bangladesh and Nepal experiencing significant unaffordable expenditures on FT (FTAR=2.7546 and FTAR=1.7960, respectively). The variation among the included countries of the SEARO Region was reflected through the wide FTAR 95% confidence intervals. 40% of the included countries from the SEARO Region experienced unaffordable expenditures on the cheapest (top-three selling) FT.

The AFRO Region, the only region associated with low-income countries in the sample and a significant proportion of lower middle-income countries (50%), was subject to unaffordable expenditures on FT (*S1 Table*). An average lowest-paid unskilled government worker needed 4.08 (95% CI 2.54 to 5.62) days of work to purchase 182.5g of the cheapest (top-three selling) FT, on average. Benin had the highest reported FTAR across the sample of all included countries in the study (=11.8159). Purchasing FT was affordable in only three of 18 included countries from the AFRO region, i.e. Mauritius (FTAR=0.3249), the Central African Republic (FTAR=0.7821) and Senegal (FTAR=0.9108). 83% of the included countries from the AFRO Region experienced unaffordable expenditures on the cheapest (top-three selling) FT.

The findings with respect to the EMRO Region are uncertain, as data from only 3 countries (of 22 in the Region) were compiled and analysed (*S1 Table*). From the available sample, an average lowest-paid unskilled government worker needed 1.22 (95% CI 0.35 to 2.10) days of work to purchase the annual recommended amount of the cheapest (top-three selling) FT (i.e., 182.5 g). In addition, there was a variation with respect to the FTARs of the included countries from the EMRO Region, with the 95% FTAR confidence intervals being wide. Purchasing FT was affordable in only one of three included countries from the EMRO Region, i.e., Lebanon (FTAR=0.5217). 67% of the included countries from the EMRO Region experienced unaffordable expenditures on the cheapest (top-three selling) FT.

Whereas FT affordability across WHO Regions was heterogeneous, no significant variations in the price per gram of the cheapest (top-three selling) FT between the WHO Regions existed. The two-sample t-tests, which considered all possible pairs of WHO Regions, concluded that there was insufficient evidence to reject the hypothesis that the mean price per gram of the cheapest (top-three selling) FT was the same in a given pair of two WHO Regions (*Supplementary Material 4)*. The only exception was the comparison of the mean price/g of the cheapest (top-three selling) FT between any WHO Region and the EMRO Region. However, this finding is inconclusive because of the limited sample size of the included countries from the EMRO Region.

**S1 Table. Descriptive Statistics by WHO Regions, in FTARs**
