# Supplementary material for: Affordability of essential medicines: The case of fluoride toothpaste in 78 countries
Source: PLoS One. 2022 Oct 19;17(10):e0275111. doi: 10.1371/journal.pone.0275111 (PMC9581416; doi:10.1371/journal.pone.0275111)
Supplement: S4 File — (DOCX) [file pone.0275111.s008.docx]

**Supplementary Material 4. Differences in mean price/g of the cheapest (top-three selling) FTs among World Bank Income Groups and WHO Regions**

To check the hypothesis that the difference in the mean price/g of the cheapest (top-three selling) FTs among World Bank Income Groups or WHO Regions was significant, two-sided hypothesis testing of the difference of mean price/g of the cheapest (top-three selling) FTs was applied to all possible pairs of Income Groups or WHO Regions, respectively. The null hypothesis of no significant difference in the mean prices/g of the cheapest (top-three selling) FTs between a given Income Group or WHO region (indexed by 1) and another Income Group or WHO region (indexed by 2),

H_0_: p_1il_-p_2il_=0

was tested against the two-sided alternative hypothesis that the difference in the mean prices/g of the cheapest (top-three selling) FTs was significant between a given pair of Income Group or WHO region,

H_1_: p_1il_-p_2il_#0

, where p_1il_ is the mean price/g of the cheapest (top-three selling) FTs in Income Group or WHO Region, indexed by 1, and p_2il_ is the proportion of the mean price/g of the cheapest (top-three selling) FTs in the other Income Group or WHO Region, indexed by 2, for a given i=0, 1,.., 6 combination of Income Groups or l= 0, 1,…, 15 combination of WHO Regions. For each combination of i or l, the test-statistic (t_il_) under the null hypothesis follows a t-distribution with

v= n_1_ + n_2_ – 2 degrees of freedom:

t_il_= $\frac{p1il-p2il}{sil\sqrt{(\frac{1}{n1}+\frac{1}{n2})}}$ ~t_v_

, where n_1_ is the number of observations of Income Group 1 or WHO Region 1, n_2_ is the number of observations of Income Group 2 or WHO Region 2, s_il_=$\sqrt{(\frac{\left( n1-1 \right){s1}^{2}}{n1}+\frac{\left( n2-1 \right){s2}^{2}}{n2})}$ is the pooled standard deviation of a given combination of i or l, s_1_^2^ is the variance of prices of the cheapest (top-three selling) FT associated with Income Group 1 or WHO Region 1 in a given combination of i or l, and s_2_^2^ is the variance of prices of the cheapest (top-three selling) FT associated with Income Group 2 or WHO Region 2 in a given combination of i or l.

If the absolute value of t_il_ is greater than the critical t-value (t_va_) at a=0.05 significance level for and v= n_1_ + n_2_ – 2 degrees of freedom for a given combination of i or l, i.e. | t_il_ |> t_va_, the null hypothesis of no significant difference in average prices between a given pair of Income Groups or WHO Regions is rejected at the 2.5% significance level. Instead, if | t_il_ | < t_va_, there is no sufficient evidence to reject the null hypothesis of no difference in average prices between a given pair of Income Groups or WHO Regions at the 2.5% level.

**S2 Table. Results from two-sample-t-tests of the difference in mean prices/g of the cheapest (top-three selling) FTs between all pairs of World Bank Income Groups and WHO Regions**
